# Chemoselective reduction and oxidation of ketones in water through control of the electron transfer pathway

**DOI:** 10.1038/srep10366

**Published:** 2015-05-28

**Authors:** Sun Min Kim, Ho Sung Yoo, Hideo Hosono, Jung Woon Yang, Sung Wng Kim

**Affiliations:** 1 Department of Energy Science, Sungkyunkwan University, Suwon 440-746, Republic of Korea; 2 IBS Centre for Integrated Nanostructure Physics, Institute for Basic Science, Suwon, Republic of Korea; 3 Materials Research Center for Element Strategy, Materials and Structures Laboratory, and Frontier Research Center, Tokyo Institute of Technology, 4259 Nagatsuta, Midori-ku, Yokohama 226-8503, Japan; 4 ACCEL Project, Japan Science and Technology Agency, 4-1-8 Honcho, Kawaguchi, Saitama 332-0012, Japan

## Abstract

The selective synthesis of different products from the same starting materials in water, which is the most abundant solvent in nature, is a crucial issue as it maximizes the utilization of materials. Realizing such reactions for ketones is of considerable importance because numerous organic functionalities can be obtained via nucleophilic addition reactions. Herein, we report chemoselective reduction and oxidation reactions of 1,2-diketones in water, which initiates anionic electron transfer from the inorganic electride [Ca_24_Al_28_O_64_]^4+^·4e^−^, through controlling the pathway of the electrons to substrates. The generation of different radical species for transient intermediates was the key process required to control the reaction selectivity, which was achieved by reacting the anionic electrons with either diketones or O_2_, leading to the formation of ketyl dianion and superoxide radicals in the reduction and oxidation reactions, respectively. This methodology that utilizes electrides may provide an alternative to the pulse radiolysis of water in synthetic chemistry.

Water, which is the most versatile substance in nature, has attracted public and scientific interest in diverse fields, such as biology, physics, chemistry and materials science, because of its ecological and economic importance for life^1^,^2^,^3^,^4^. Following nature’s chemical lead, considerable efforts have focused on using water as a solvent in synthetic organic chemistry for the development of practical reactions^5^,^6^,^7^,^8^. Although the use of water as the solvent in organic reactions has been restricted by hydrolysis reactions^9^,^10^, the discovery of Diels-Alder reactions^11^ triggered the widespread use of water as an alternative solvent for replacing typical organic solvents, such as THF, toluene and alcohols, and for enhancing the rates and selectivities of reactions^12^,^13^,^14^. The myriad of uses of water in organic chemistry is attributed to the characteristic features of water, including its hydrophobic, hydrogen bonding and polarity effects, which accelerate electron transfer as one of the key processes in water-mediated reactions^15^,^16^.

The investigation of electron transfer in aqueous solutions was motivated by the discovery of solvated electrons during the pulse radiolysis of water, which is a well-established technique for the generation of electrons in water^17^,^18^. However, the short lifetime of solvated electrons and the generation of highly reactive radical species (e.g., ·OH, ·O^2−^, ·O_2_H, etc.) have hindered the widespread use of water in synthetic chemistry due to the degradation of both the reactivity and selectivity of the reactions^19^. Furthermore, the generation of excess electrons and radicals reduces selective electron transfer to organic substrates or induces over-reaction due to multiple electron transfer^20^. Thus, in water-mediated organic reactions, control over the electron transfer pathway is crucial for designing reactions with high yields and selectivities.

The development of chemoselective organic transformations is one of the most fundamental tasks in synthetic chemistry because it reduces material consumption, superfluous trimming and re-functionalization steps in reactions^21^,^22^. In particular, achieving chemoselectivity in water-mediated reactions by controlling the electron transfer pathway enables a single reactant to be transformed into structurally diverse products with different functionalities. However, the chemoselectivity of fundamental reactions in water, such as oxidation and reduction, has not been previously explored. Herein, we report chemoselective reactions both reduction and oxidation of 1,2-diketones in water by controlling the intermolecular electron transfer pathway with inorganic [Ca_24_Al_28_O_64_]^4+^∙4e^−^ electrides.

Electrides are ionic crystals with cavity-trapped electrons that act as anions^23^. The first electride was synthesized in 1983 using crown ethers and solvated electrons in alkali-ammonia solutions to provide precursors for anionic electrons^24^. Because the organic electrides were chemically and thermally unstable and decomposed in ambient conditions, their applications for synthetic chemistry were limited^25^. In 2003, the first electride stable at room temperature was synthesized using the inorganic complex oxide 12CaO·7Al_2_O_3_ to form [Ca_24_Al_28_O_64_]^4+^∙2O^2−^, which has a positively charged [Ca_24_Al_28_O_64_]^4+^ framework composed of 12 subnanometer-sized cages and two O^2−^ ions as counteranions^26^. Because of the anionic electrons, the [Ca_24_Al_28_O_64_]^4+^∙4e^−^ electride exhibited unique properties, such as improved electron transfer performance due to its low work function (~2.4 eV)^27^ and transformation to a molten state at 1873 K while maintaining the solvated electrons^28^. This inorganic electride has recently appeared in a new class of reagents that serves as electron generators in aqueous solutions, facilitating the pinacol coupling reaction of aldehydes^29^. One consequence of the reaction between the [Ca_24_Al_28_O_64_]^4+^∙4e^−^ electride and water is that when electrides are dissolved in water, the anionic electrons that are loosely trapped in cavities can be released and directly transferred to the reactants, thus facilitating chemical reactions. Applying [Ca_24_Al_28_O_64_]^4+^∙4e^−^ electrides to water-mediated chemoselective reactions of ketones, which are ubiquitous functional groups, can provide important insights into the fundamental roles of electrons in water-mediated synthetic organic reactions and a broad opportunity for producing diverse functional molecules.

## Results and discussion

**Figure 1:**
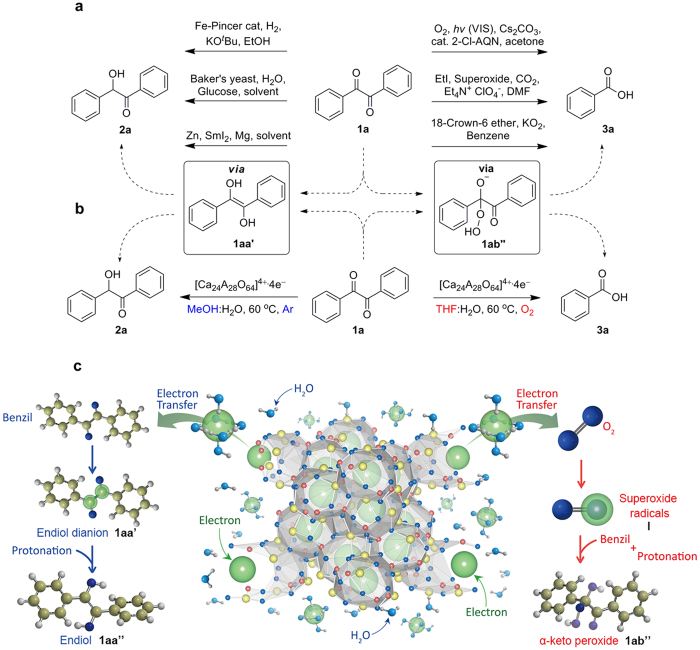
Strategies for the chemoselective reduction and oxidation of 1,2-diketones using the [Ca_24_Al_28_O_64_]^4+^∙4e^−^ electride. (**a**) Previous reports on reduction and oxidation reactions of benzil via the electron transfer process. (**b**) Present work on the chemoselective reduction and oxidation of benzil using the [Ca_24_Al_28_O_64_]^4+^∙4e^−^ electride. (**c**) Schematic illustration of the chemoselective reduction and oxidation of benzil by controlling the electron transfer pathway to benzil or O_2_ via the release of electrons from the [Ca_24_Al_28_O_64_]^4+^∙4e^−^ electride via hydrolysis as well as the possible solvation of electrons by water molecules.

Figure 1 describes the strategy for realizing chemoselective oxidation and reduction reactions of ketones in water through controlling both the electron transfer pathway and the formation of different intermediates in each reaction. When the targeted electron acceptor is benzil **1a**, which is the model substrate selected for incorporating an electron acceptor (such as a carbonyl moiety), the path of the reaction is primarily dependent on the type of catalysts and reagents being used^30^,^31^,^32^,^33^,^34^,^35^,^36^,^37^. In fact, either the oxidation or reduction reaction of benzil **1a** can occur by using different reagents that completely alter the formation of intermediates to result in enediol **1aa”** in reduction reactions and α-keto peroxide **1ab”** in oxidation reactions (Fig. 1a). However, despite extensive research on the reaction of benzil with diverse electron transfer reagents and solvents, there was no success in utilizing both electron transfer pathways with one reagent in a highly selective manner. Furthermore, a selective reaction in the presence of water is difficult to achieve because of the vigorous water splitting reactions that occur between the excess electrons from the reagents and water. Nevertheless, as shown in Fig. 1b, it is hypothesized that the chemoselective reaction of benzil **1a** in the presence of water is possible if the electrons are smoothly generated through a moderate cage-opening rate by the robustness of the [Ca_24_Al_28_O_64_]^4+^∙4e^−^ electride in water, which diminishes the loss of electrons by water splitting. The solvated electrons would then react with different substances, such as benzil or O_2_, to selectively form the transient radicals (**1aa’** or **I**). To realize our hypothesis, the [Ca_24_Al_28_O_64_]^4+^∙4e^−^ electride, which is an electron-donating reagent that possesses moderate reactivity in water, was used because of its smooth electron release and the possible solvation of anionic electrons by water molecules.

Figure 1c shows the details of our approach of using the [Ca_24_Al_28_O_64_]^4+^∙4e^−^ electride to realize chemoselective reactions of benzil in water. In water-mediated reactions with the [Ca_24_Al_28_O_64_]^4+^∙4e^−^ electride, water has two important roles: 1) releasing anionic electrons from the electrides and 2) acting as the medium for transferring the anionic electrons to electron acceptors. The cage structure of the [Ca_24_Al_28_O_64_]^4+^∙4e^−^ electride slowly decomposes in water and forms a Ca-Al-O-OH gel as aluminous cement, leading to the release of anionic electrons. The released electrons are transferred to the appropriate electron acceptors, most likely through electron solvation by water molecules, which are aptly referred to as solvated electrons. During the reaction, we observed the evolution of hydrogen gas as a result of water splitting by electrons, which indicates that electrons are in proximity to water molecules. Because the formation of transient radicals is critical in determining the course of reactions and producing an intermediate, controlling the electron transfer pathway is the major concern. In the reduction reaction, benzil **1a** accepts two electrons and then transforms into the enediol dianion **1aa’**. In postulating the oxidation reaction, we intentionally involved oxygen molecules in the reaction to facilitate the formation of the superoxide radicals **I**.

Table 1 presents the results of the reduction reaction of benzil **1a**, which was conducted under an inert argon atmosphere to prevent the incorporation of oxygen molecules in the reactions. Benzil **1a** accepts two electrons from the [Ca_24_Al_28_O_64_]^4+^∙4e^−^ electride and then transforms into the enediol dianion **1aa’**. The resulting enediol dianion species may subsequently undergo protonation, affording an α-hydroxy ketone via keto*-*enol tautomerization. To identify the necessity of a proton source, we initially performed the reaction of benzil **1a** with 1.0 equivalent of electride in water alone. However, no reaction occurred, most likely due to the poor solubility of benzil **1a** in water (Table 1, entry 1), which prompted the use of co-solvent to improve the solubility of benzil **1a**. We observed the reaction progress when deoxygenated THF-H_2_O (v/v = 1:1) was applied as a co-solvent to the reaction of benzil **1a** with 1.0 equivalent of [Ca_24_Al_28_O_64_]^4+^∙4e^−^ at 60 °C under an inert argon atmosphere. In this reaction, benzil **1a** was reduced to benzoin **2a** in low yield after 24 h (Table 1, entry 2). The use of an additional equivalent of electride led to a slight improvement in the reaction yield (Table 1, entry 3).

A significant enhancement in the yield of **2a** was achieved by replacing the THF-H_2_O co-solvent with MeOH-H_2_O co-solvent (Table 1, entry 4). This result is attributed to two reasons: 1) the difference in solvent polarity, which facilitates rapid electron transfer^38^, and 2) the difference in solvent acidity, which accelerates protonation of the products. A more polar solvent is expected to facilitate electron transfer because of the stronger coupling between molecules and the faster solvation response. Although the two solvents, MeOH and H_2_O, have similar pKa values (pKa of H_2_O = 15.7 and pKa of MeOH = 15.5), the more acidic MeOH molecule donates a proton and becomes a ^−^OMe ion. Upon acid dissociation, the methoxide ion is stabilized by the solvation of H_2_O molecules, in which the positively charged H atoms move toward the methoxide ion solutes. This process makes MeOH more acidic than H_2_O, rendering MeOH a sufficient proton source in the reduction process.

When using alcohols in the co-solvent, the yield of product **2a** increased in the following order of acidity and electron transfer rates^39^ (methanol > ethanol > isopropanol > *tert*-butanol) (Table 1, entries 4-7). Notably, the reaction time decreased as the MeOH-H_2_O volume ratio was changed from 1:1 to 1:3 (Table 1, entry 8). It is hypothesized that the increased polarity from the greater amount of water contributed to the acceleration of cage opening and donation of electrons from the electrides. Finally, in contrast to deoxygenated THF-H_2_O (v/v = 1:1) (Table 1, entry 3), the use of a partially oxygenated THF-H_2_O co-solvent yielded a small amount of benzoic acid **3a** (Table 1, entry 9), which was identified by NMR spectroscopy. This result indicated that the oxidative cleavage reaction of benzil **1a** should be investigated by introducing oxygen molecules as electron acceptors into the reaction. To verify the necessity of electrons in the reaction, we examined the reaction using [Ca_24_Al_28_O_64_]^4+^∙2O^2−^ oxide, which is the precursor of the [Ca_24_Al_28_O_64_]^4+^∙4e^−^ electride (Table 1, entry 10). No reaction took place, which strongly indicates that the electrons of the electride are essential for the reduction of 1,2-diketones. Furthermore, no reaction occurred in methanol, indicating that water is indispensable media to release and transfer the electrons via hydrolysis of cage structured [Ca_24_Al_28_O_64_]^4+^∙4e^−^ electride (Table 1, entry 11).

Following the identification of benzoic acid **3a** after using the partially oxygenated THF-H_2_O co-solvent, we changed the reaction atmosphere from inert argon gas to molecular oxygen as a terminal oxidant, which enabled an increase in the amount of oxygen molecules that was dependent upon the different solubilities of oxygen molecules in each solvent system. We initially performed the reaction of benzil **1a** with 2.0 equivalents of electride in MeOH-H_2_O (v/v = 1:3) at 60 °C under an oxygen atmosphere and obtained benzoic acid **3a** in 36% yield (Table 2, entry 1). The use of DMF, DMSO and MeCN solvent in water afforded benzoic acid **3a** in moderate yields (up to 53%; Table 2, entries 2-4). The reaction was optimized using THF-H_2_O co-solvent, which provided the best yield (96%; Table 2, entries 5-7).

Increasing the amount of THF in the co-solvent significantly improved the yield of benzoic acid **3a** (Table 2, entries 5-7), albeit with longer reaction times (20 h). This improvement may be explained by the following two reasons: i) due to the higher solubility of O_2_ in THF (2.1 mM) compared to water (0.26 mM), the reaction is accomplished through an enhanced formation of superoxide radicals, or ii) decreasing the ratio of water in the co-solvent causes a reduction in polarity, which retards cage decomposition and electron release into the solvent. The slow generation of electrons and the reduced rate of molecular oxygen permeating into the co-solvent may minimize the probability of electron loss by reaction with O_2_ rather than water.

Based on the chemoselective reactions established above, we expanded the reaction scope to the diverse set of 1,2-diketone molecules listed in Table 3. In the reactions of the unsubstituted aromatic 1,2-diketones, both the reduction and oxidation reactions afforded the corresponding products in high yields (Table 3, entries 1 and 2). Additionally, both the reduction and oxidation reactions proceeded not only for the 1,2-diketones bearing electron-withdrawing substituents on the aryl ring (Table 3, entries 3 and 4) but also for other substrates bearing strong electron-donating substituents, such as -Me or -OMe groups (Table 3, entries 5, 6 and 7). This result indicates that this electride, which possesses a low work function, has great potential for use as a powerful reducing agent in chemical reactions.

**Figure 2:**
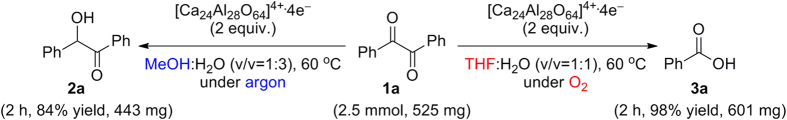
Large-scale production of **2a** and **3a** using the [Ca_24_Al_28_O_64_]^4+^∙4e^−^ electride.

We also examined the feasibility of large-scale reactions using the [Ca_24_Al_28_O_64_]^4+^∙4e^−^ electride in water to address the potential limitations of utilizing the [Ca_24_Al_28_O_64_]^4+^∙4e^−^ electride in organic synthesis. For both the reduction and oxidation reactions, benzil **1a** was used as shown in Fig. 2 on a 2.5 mmol scale (525 mg of benzil), which was 12.5 times greater than the amount used for the optimization. Notably, benzoin **2a** and benzoic acid **3a** were obtained in yields of 84% and 98%, respectively.

Based on the experimental results, we propose mechanisms associated with electron transfer from the electrides in aqueous solutions. For the mechanism of the reduction process, two electrons from the [Ca_24_Al_28_O_64_]^4+^∙4e^−^ electride are transferred to 1,2-diketone **1a**. Consequently, two ketyl radical anions of 1,2-diketone are generated and quickly converted to enediol dianion **1aa’** via radical dimerization^40^. The use of a protic solvent allowed the enediol dianion to be protonated, thereby forming enediol intermediate **1aa”** which undergoes tautomerization to form α-hydroxy ketone **2a**.

In contrast to reduction, the oxidation reaction was a rather complex process. The oxidative cleavage reaction occurs in a two-step process. In contrast to the reduction reaction that occurs via direct electron transfer to benzil **1a**, in the oxidation reaction, the electrons are firstly transferred to the oxygen molecule, generating superoxide radicals **I**^41^. These radical anions then react with benzil **1a**, leading to the formation of α-peroxy ketone intermediate **1ab”**.

**Figure 3:**
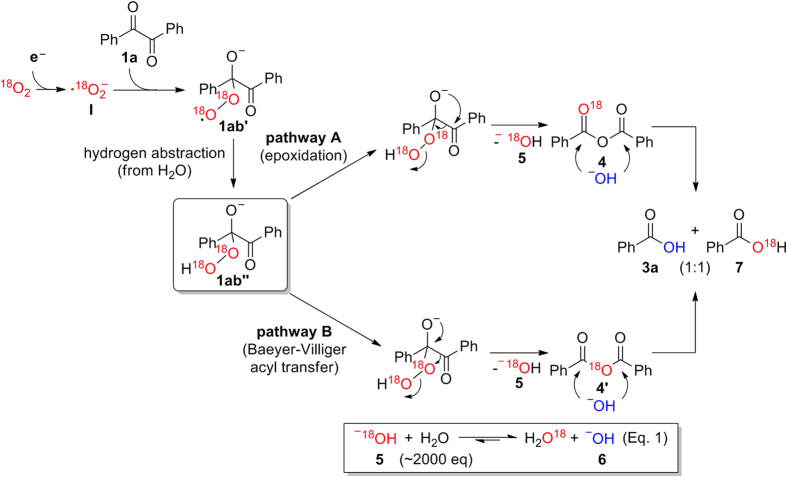
Proposed mechanism for the oxidative cleavage of 1,2-diketone utilizing using the [Ca_24_Al_28_O_64_]^4+^∙4e^−^ electride via ^18^O isotopic labeling experiments.

To obtain insight into the reaction mechanism for oxidation, we conducted ^18^O isotopic labeling experiments using ^18^O_2_ gas, which provides an integral part of the electron transfer pathway for the formation of carboxylic acids, as shown in Fig. 3. The superoxide radicals **I** was initially generated by the reaction between ^18^O_2_ and a single electron from the electride and then most likely transferred to **1a**, affording α-keto peroxy radical anion **1ab’**. Then, hydrogen abstraction from water occurred almost simultaneously to form α-keto peroxide **1ab”**. Subsequently, two different ^18^O-labeled anhydrides, **4** and **4’**, were formed via either epoxidation (pathway A) or the Baeyer-Villiger reaction with acyl group migration from α-keto peroxide **1ab”**, with simultaneous release of ^18^O-labeled hydroxide ion **5** (pathway B). Presumably, the ^18^OH^−^ ions rapidly exchanged protons with an extremely large amount of water to produce hydroxide ion (OH^−^) **6** according to Le Chatelier’s principle (Eq. 1). The nucleophilic attack of a hydroxide ion on the carbonyl carbon of ^18^O-labeled anhydrides **4** and **4’** produced ^18^O-labeled benzoic acid **7** and ^16^O-incorporated benzoic acid **3a** in equal quantities.

**Figure 4:**
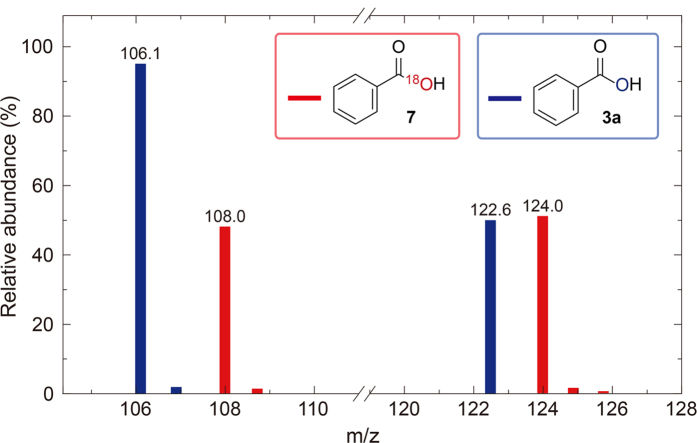
^18^O isotopic labeling results of the oxidative cleavage reaction of 1,2-diketone.

To support the proposed mechanism, the resulting products (3a and 7) were subjected to gas chromatography-mass spectrometry (GC-MS) for isotopic analysis. The ^16^O and ^18^O compositions of the individual carboxylic acids were determined based on the relative abundances of mass peaks at m/z = 122 for ^16^O and m/z = 124 for ^18^O. As shown in Fig. 4, it is clear that the relative abundances of the isotopic masses for each product are identical to those of the isotopic masses. This result strongly suggests that oxygen molecules are incorporated into 1,2-diketone, producing carboxylic acids, which is initiated by the formation of superoxide radicals **I**.

**Figure 5:**
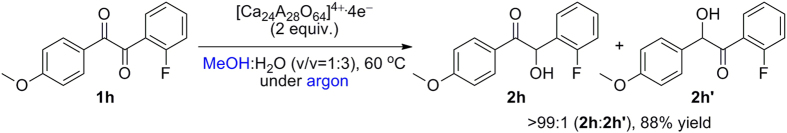
Regiospecific reduction of unsymmetrical 1,2-diketone.

To expand the synthetic application of this selective fashion, we then investigated the regioselectivity in the reaction of unsymmetrical 1,2-diketone **1h** with the electride (Fig. 5). The most favourable reduction site was the carbonyl adjacent to the electron-withdrawing substituted aromatic ring, showing the specific regioselectivity of the products in a >99: 1 ratio of **2h : 2h′**.

## Summary

The use of the [Ca_24_Al_28_O_64_]^4+^∙4e^−^ electride, an exceptional electron source, allowed the first demonstration of chemoselective oxidation and reduction reactions with ketones in water. Transient radical formation was integral to the chemoselective success of the reactions, and the formed radical was dependent on either the presence of a proton source or an oxygen molecule in each electron transfer process. This proposed mechanism explains why the electron transfer process in water, which is omnipresent but often goes unnoticed, should be regarded as a critical mechanistic step for the formation of intermediate substances in chemical reactions. Additionally, this new methodology using the [Ca_24_Al_28_O_64_]^4+^∙4e^−^ electride as a solid-state reducing agent in water may provide an alternative to the high-energy pulse radiolysis of water, in which it is not possible to realize these selective chemical reactions. Finally, the solvated electrons, which are extremely potent reducing agents, can be used for oxidation reactions when the electron pathway is controlled to form the proper transient intermediates according to the desired reaction conditions.

## Methods

### **Typical procedure for reduction of benzil using C12A7:e** ^−^

In 20 mL vial, C12A7:e^−^ (362 mg, 0.5 mmol) was added to solution of benzil (42 mg, 0.2 mmol) in 8 mL of EtOH:H_2_O (v/v = 1:3) co-solvent at room temperature. The reaction was vigorously stirred for 2 h, then quenched with 5% HCl solution, and extracted with EtOAc (3 × 2 mL). The combined organic layers were dried over MgSO_4_. The residue was purified by flash column chromatography on silica gel (hexanes/ethyl acetate = 4:1 to 2:1) to give benzoin (38 mg, 91% yield).

### **Typical procedure for oxidative cleavage reaction of benzil using C12A7:e** ^**−**^

In 20 mL vial, C12A7:e^−^ (362 mg, 0.5 mmol) was added to solution of benzil (42 mg, 0.2 mmol) in 8 mL of THF:H_2_O (v/v = 1:1) co-solvent at room temperature. The reaction was vigorously stirred for 12 h, then quenched with 5% HCl solution, and extracted with EtOAc (3 × 2 mL). The combined organic layers were dried over MgSO_4_. The residue was purified by flash column chromatography on silica gel (hexanes/ethyl acetate = 4:1 to 1:1) to give benzoic acid (46 mg, 96% yield).

### ^**18**^ **O-labelling experiments by GC/MS**

The ratios of ^16^O^16^O, ^16^O^18^O and ^18^O^18^O were determined based on the intensities of mass peaks at m/z = 122, 124 and 126, analysed by a Varian GC-MS 4000 gas chromatograph equipped with mass spectrometer at room temperature.

## Additional Information

**How to cite this article**: Kim, S. M. *et al.* Chemoselective reduction and oxidation of ketones in water through control of the electron transfer pathway. *Sci. Rep.* **5**, 10366; doi: 10.1038/srep10366 (2015).

## Figures and Tables

**Table 1:**
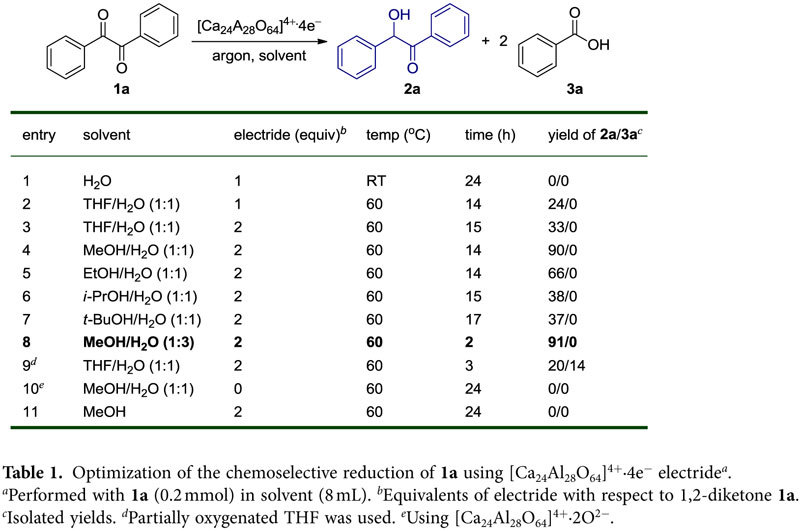
Optimization of the chemoselective reduction of **1a** using [Ca_24_Al_28_O_64_]^4+^∙4e^−^ electride^*a*^.

**Table 2:**
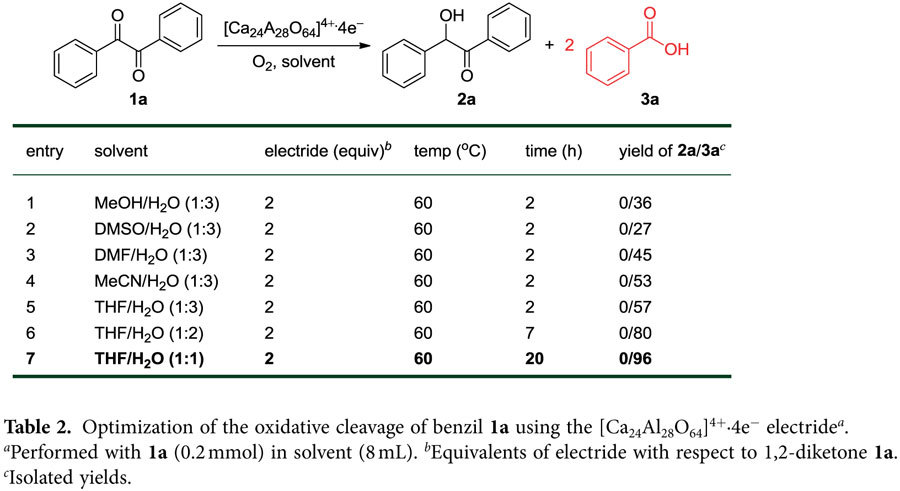
Optimization of the oxidative cleavage of benzil **1a** using the [Ca_24_Al_28_O_64_]^4+^∙4e^−^ electride^*a*^.

**Table 3:**
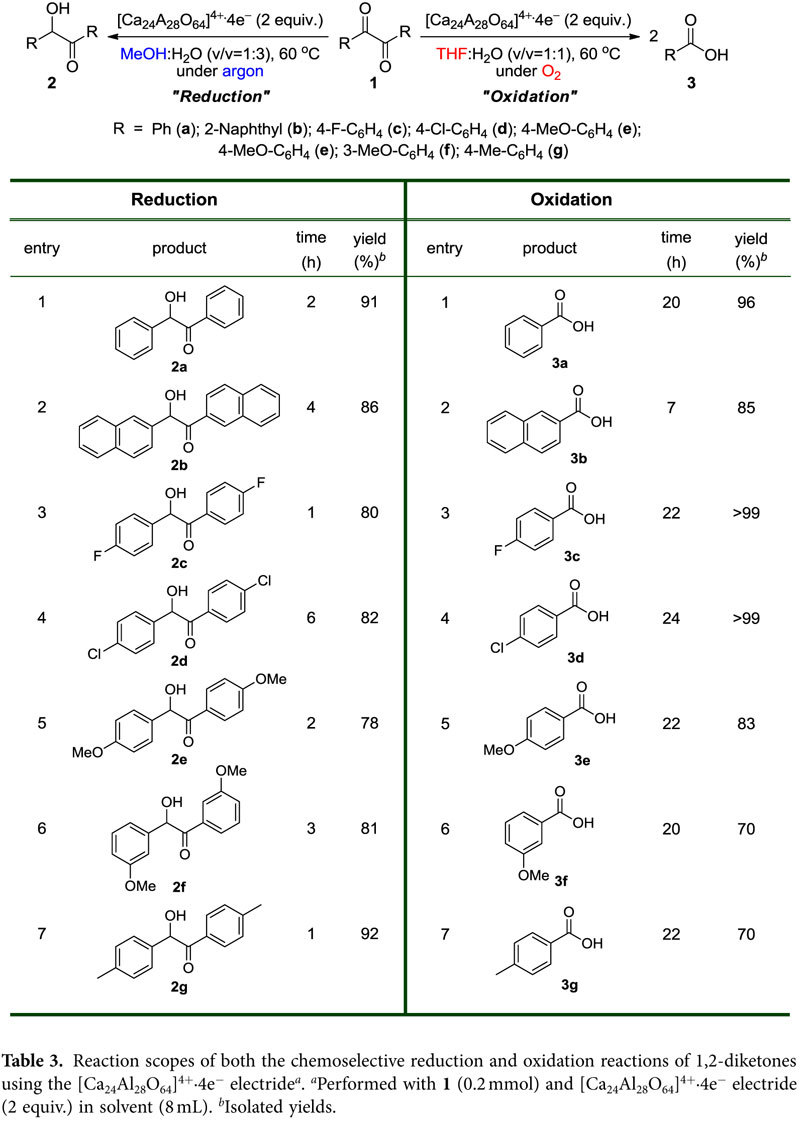
Reaction scopes of both the chemoselective reduction and oxidation reactions of 1,2-diketones using the [Ca_24_Al_28_O_64_]^4+^∙4e^−^ electride^*a*^.
